# Oral keratinocytes support non-replicative infection and transfer of harbored HIV-1 to permissive cells

**DOI:** 10.1186/1742-4690-5-66

**Published:** 2008-07-17

**Authors:** Anjalee Vacharaksa, Anil C Asrani, Kristin H Gebhard, Claudine E Fasching, Rodrigo A Giacaman, Edward N Janoff, Karen F Ross, Mark C Herzberg

**Affiliations:** 1 Department of Diagnostic and Biological Sciences, School of Dentistry, University of Minnesota, Minneapolis, MN 55455, USA; 2 Mucosal and Vaccine Research Center, Minneapolis VA Medical Center, Minneapolis, MN 55417, USA; 3 Division of Infectious Diseases, Colorado Center for AIDS Research, and the Mucosal and Vaccine Research Program Colorado, University of Colorado Denver, and the Denver Veterans Affairs Medical Center, Denver, CO 80220, USA

## Abstract

**Background:**

Oral keratinocytes on the mucosal surface are frequently exposed to HIV-1 through contact with infected sexual partners or nursing mothers. To determine the plausibility that oral keratinocytes are primary targets of HIV-1, we tested the hypothesis that HIV-1 infects oral keratinocytes in a restricted manner.

**Results:**

To study the fate of HIV-1, immortalized oral keratinocytes (OKF6/TERT-2; TERT-2 cells) were characterized for the fate of HIV-specific RNA and DNA. At 6 h post inoculation with X4 or R5-tropic HIV-1, HIV-1*gag *RNA was detected maximally within TERT-2 cells. Reverse transcriptase activity in TERT-2 cells was confirmed by VSV-G-mediated infection with HIV-NL4-3Δenv-EGFP. AZT inhibited EGFP expression in a dose-dependent manner, suggesting that viral replication can be supported if receptors are bypassed. Within 3 h post inoculation, integrated HIV-1 DNA was detected in TERT-2 cell nuclei and persisted after subculture. Multiply spliced and unspliced HIV-1 mRNAs were not detectable up to 72 h post inoculation, suggesting that HIV replication may abort and that infection is non-productive. Within 48 h post inoculation, however, virus harbored by CD4 negative TERT-2 cells *trans *infected co-cultured peripheral blood mononuclear cells (PBMCs) or MOLT4 cells (CD4+ CCR5+) by direct cell-to-cell transfer or by releasing low levels of infectious virions. Primary tonsil epithelial cells also *trans *infected HIV-1 to permissive cells in a donor-specific manner.

**Conclusion:**

Oral keratinocytes appear, therefore, to support stable non-replicative integration, while harboring and transmitting infectious X4- or R5-tropic HIV-1 to permissive cells for up to 48 h.

## Introduction

During oral-sexual contacts and breast feeding, oral keratinocytes of the stratified squamous epithelium represent the most abundant cell type exposed to infectious HIV-1 [1-5]. Since HIV-1^gag ^RNA is detected in cytokeratin-positive cells of mucosal biopsies [6] and shedding buccal cells [7], HIV-1 could infect and persist in oral keratinocytes during primary infection or secondary to systemic dissemination. HIV-1 that is harbored in keratinocytes could be transferred to proximal immature dendritic (Langerhans) cells of the mucosal epithelium. These Langerhans cells present HIV-1 to permissive CD4+ T lymphocytes. Alternatively, permissive lymphoid cells could access virus at inter-epithelial spaces where HIV-1 particles have been visualized by electron microscopy [7].

In infant [8,9] and adult primates [10], cell-free simian immunodeficiency virus (SIV) infects intact oral mucosa within one day after non-traumatic exposure and viral RNA is detected in the proximal epithelium. About four days later, signs of SIV infection appear in the gut, followed by viremia and simian AIDS. Hence, the pathogenesis of SIV-infection in primates is consistent with the possibility that clinical exposures of HIV-1 to the oral and oropharyngeal mucosa result in primary infections of the keratinocytes in the squamous epithelium. Primary human infections from an oral epithelial focus, therefore, could result in systemic dissemination of HIV-1.

Oral keratinocytes use an atypical mechanism to facilitate entry of HIV-1. In permissive cells, which express CD4, HIV-1 efficiently enters cells using gp120-mediated membrane fusion [11-13]. Since oral keratinocytes do not express CD4 [14], HIV-1 entry into keratinocytes is expected to be less efficient than other permissive cells. Galactosylceramide (GalCer) [15] and heparin sulfate proteoglycans (HSPGs) [16,17] have been suggested to be alternate receptors for HIV-1 on CD4-negative cells including keratinocytes, enabling HIV-1 to enter host cells in an envelope-independent manner [18]. After internalization, HIV-1 may be mobilized intracellularly by selective and rapid transcellular vesicular trafficking [19].

Based on *in vitro *studies, it is unclear if HIV-1 replicates in oral keratinocytes or if the cells harbor and transfer infectious particles (*trans *infect) to permissive cells such as peripheral blood mononuclear cells [20-22]. Suggestive of viral integration, HIV-1^LTR/gag ^DNA has been isolated from primary gingival keratinocytes [20], but HIV-1^LTR/gag ^PCR primers could have amplified unintegrated linear HIV-1 DNA. HIV-1 propagated in permissive producer cells is contaminated by integrated human HIV-1 DNA sequences [23]. These sequence contaminants are potentially mistaken for new integration events when detected by PCR. To remove contaminating DNA, HIV-1 has been treated with DNase before infection of keratinocytes, but the efficacy of this approach was not reported [24]. Other studies of oral keratinocytes [20-22] have not reported expression of integrated HIV DNA or two-LTR circles [25].

To determine the fate of HIV-1 in oral keratinocytes, we investigated key life cycle events reported in permissive cells [26,27], including viral entry, integration, and the expression of HIV-specific genes. To eliminate interpersonal variability that can confound studies of primary cells in culture, we studied immortalized OKF6/TERT-2 (TERT-2) cells as a genetically and phenotypically consistent oral keratinocyte [28] target for HIV-1 infection. Originally isolated from the floor of a human mouth, TERT-2 cells show a normal phenotype and an extended replicative life span [28]. We hypothesized that HIV could integrate and replicate in TERT-2 oral keratinocytes, produce sufficient HIV-1 to infect neighboring permissive cells, and that key steps in the life cycle are demonstrable. Since receptive transmission by an oral route occurs infrequently [29], HIV-1 infection and viral production were expected to be of low abundance in TERT-2 cells. To show convincingly that HIV-1 integrates into the genome of keratinocytes, albeit at low levels, highly sensitive nested PCR was utilized. To eliminate contaminating integrated human HIV-1 DNA sequences derived from producer cells, genomic DNA was isolated directly from the nuclei of HIV-1 inoculated TERT-2 cells and the fate of HIV-specific RNA was followed over time.

## Results

### Oral keratinocytes capture and transfer HIV-1 to infect peripheral blood mononuclear cells

**Figure 1:**
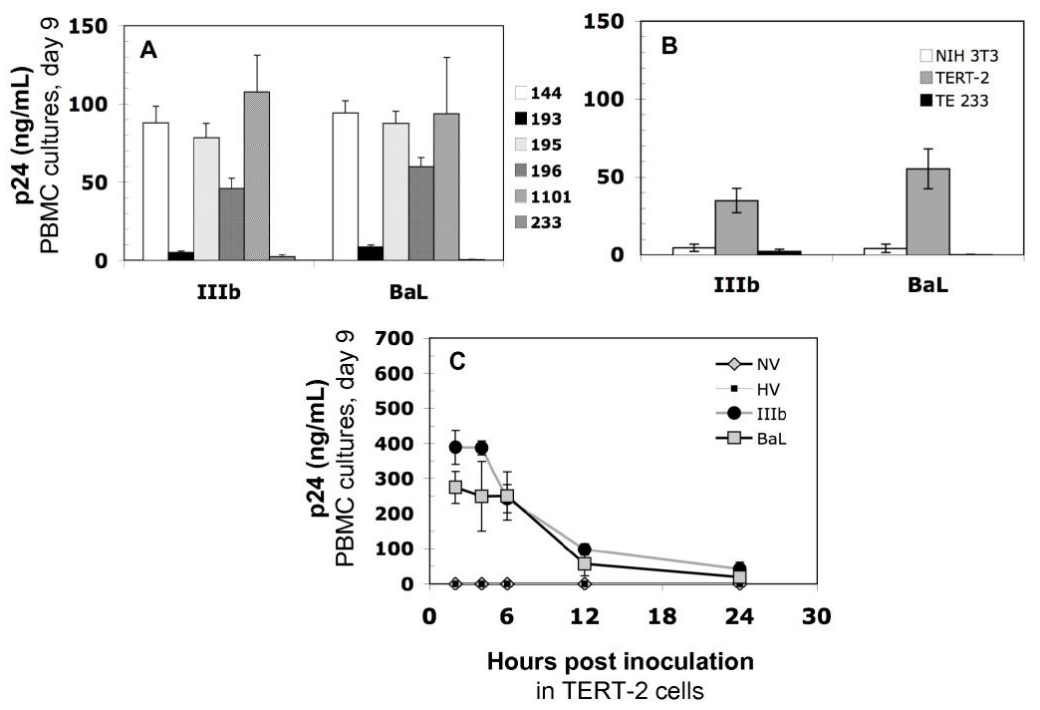
**Oral keratinocytes *trans *infect HIV-1 to permissive PBMCs**. TERT-2 or TE monolayers were inoculated and incubated for 6 h with lab-adapted HIV-1, IIIb or BaL. Tonsils were obtained from six donors (tissues 144, 193, 195, 196, 1101, and 223). Cells from each donor were propagated separately and TE cells were cultured as described in Materials and Methods. After incubation, cells were trypsinized, washed to remove non-internalized particles, and then co-cultured with PHA-activated PBMCs (2 × 10^5 ^cells) in PBMC growth media. To estimate HIV-1 *trans *infection from keratinocytes, PBMCs supernatants were collected on day 9 post inoculation and p24^gag ^expression was estimated using ELISA. (A) TE cells from each donor differentially *trans *infect HIV-1 to PBMCs. (B) TERT-2 and TE 223 cells were tested side-by-side in the same experiments to compare HIV uptake and transfer. Mouse fibroblast cells (NIH 3T3) were included as a negative control. (C) To investigate the rate of HIV-1 *trans *infection over time, TERT-2 cells were trypsinized and washed to remove extracellular HIV-1 at indicated times post inoculation. TERT-2 cells from each time point were then co-cultured with PBMCs and p24^gag ^production was analyzed. TERT-2 cells incubated with media only (no virus; NV) or heat-inactivated HIV-1 BaL (HV) were included as negative controls. Data in panel A represent the mean ± standard deviation of triplicate determinations in one experiment since the availability of primary tonsil cells from each donor was limited. Data in panel B and C are reported as the mean ± standard deviation from three independent experiments each performed in triplicate.

Primary tonsil epithelial (TE) cells from six donors were compared for the ability to transfer (*trans *infect) HIV-1 to peripheral blood mononuclear cells (PBMCs) *in vitro *(Fig. 1A). After incubation with HIV-1 (IIIb or BaL) for 6 h, TE cells from some donors (nos. 144, 195, 196, and 1101) appeared to capture and transfer the lab-adapted HIV strains; exceptions included TE cells from donor tissues 193 and 233 (Fig. 1A). To avoid the subject-to-subject variability seen in primary TE cell cultures, we evaluated TERT-2 cells for further study of capture, infection, replication and transfer of HIV-1 to permissive cells.

TERT-2 cells appeared to transfer both HIV-1 strains to PBMCs (Fig. 1B), with average effectiveness when compared to the TE cells from different donors (Fig. 1A). Performed in parallel with TERT-2 cells, *trans *infection by TE cells (tissue no. 233) was consistent with the previous experiment and similar to non-permissive mouse fibroblasts (NIH 3T3) (Fig. 1B). At similar levels to TERT-2 cells, several other keratinocyte cell lines, including TR146 [30] and KB [31], also *trans *infected HIV-1 IIIb and BaL to permissive cells (data not shown).

To determine the time course of uptake and transfer, HIV-1 was incubated with TERT-2 cells (MOI 0.01), trypsinized to remove extracellular virus, and co-cultured with PBMCs at indicated times for up to 24 h. After incubation with HIV-1 for up to 6 h, TERT-2 cell internalized HIV-1 appeared to be maximally transferable to PBMCs. *Trans *infection of internalized HIV-1 from TERT-2 cells decreased to the limits of detection by 24 h post inoculation (Fig. 1C).

### Putative HIV receptor expression on TERT-2 oral keratinocytes and TE primary cells

Since oral keratinocytes are negative for CD4 [21], we analyzed TERT-2 cells for alternative HIV receptors and co-receptors by flow cytometry (Table 1) and immunofluorescence staining (data not shown). In preliminary experiments, candidate molecules of interest were cleaved from TERT-2 cells when harvested using trypsin (data not shown). Consequently, TERT-2 cells were harvested without trypsin for flow cytometry analysis. TERT-2 cells were negative for CD4 as expected (Table 1) and 80% of the cells were positive for CD104, a β4-integrin chain generally expressed by epithelial cells [32] (Table 1). TERT-2 cells were also positive for HSPGs (91 ± 1%) and less frequently positive for the HIV-1 co-receptor CXCR4 (3.5 ± 2%) and galactosylceramide (GalCer) (<1%). Unlike salivary gland epithelial cells [20], less than 1% of TERT-2 cells expressed the CCR5 co-receptor for HIV-1. TE cells (tissue nos. 164, 193 and 196) were also analyzed for putative HIV-1 receptors and co-receptors (Table 1). TE cells did not express CD4, or CXCR4 or CCR5 (< 1%). When compared to TERT-2 cells, TE cells express GalCer (4 ± 0.1%) with similar frequency, but HSPGs (13 ± 7%) are expressed less frequently.

**Table 1:** Putative HIV receptor expression on oral keratinocytes

**Receptor**	**Function**	**TE ** (Mean ± SD)^a^	**TERT-2 ** (Mean ± SD)^b^
CD104 (β4 integrin)	transmembrane protein expressed predominantly in epithelial cells [32]	80 ± 11	83 ± 4
HSPGs	HIV gp120 binding [70]	13 ± 7	91 ± 20
GalCer	HIV gp120 binding [71]	4 ± 0.1	< 1
CD4	HIV gp120 binding [72]	< 1.0	< 1
CXCR4	X4-tropic chemokine co-receptor [73]	< 1.0	3.5 ± 2
CCR5	R5-tropic chemokine co-receptor [73]	< 1.0	< 1
CD3, CD11a/LFA-1, CD32, CD64, CD89, DC-SIGN, Macrophage Mannose Receptor		< 1.0	Not tested
Human fibroblast		4 ± 4	Not tested

^a ^Mean ± SD of four independent experiments (1 experiment from tissue no. 164 and 193, and 2 experiments from tissue no. 196)

^b ^Mean ± SD of three independent experiments

### TERT-2 oral keratinocytes support HIV-1 reverse transcription and integration

**Figure 2:**
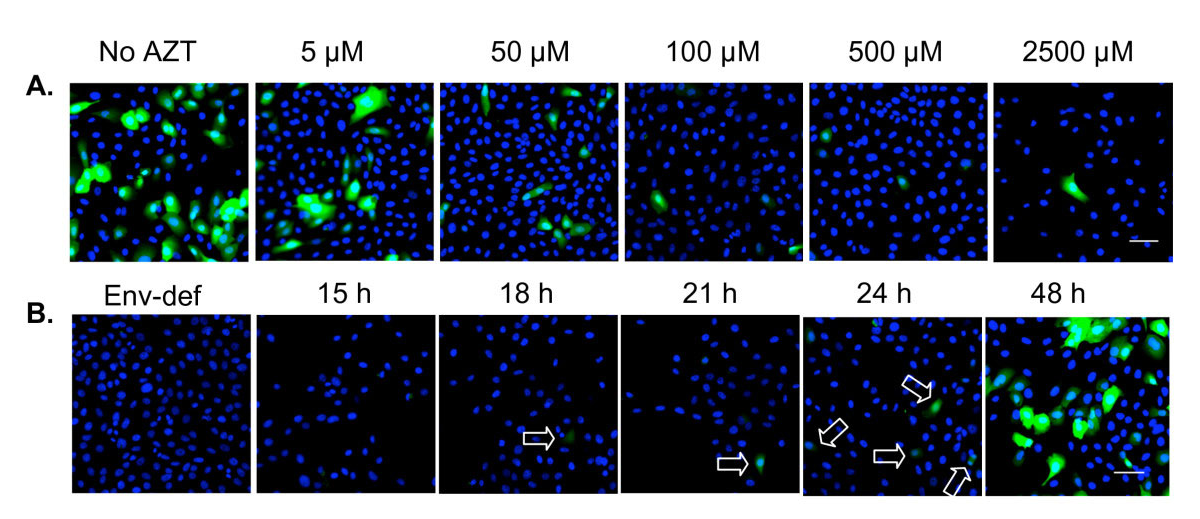
**Replication-incompetent HIV-NL4-3Δenv virus-like particles infect TERT-2 keratinocytes**. Replication-incompetent HIV-NL4-3Δenv virus-like particles (VLPs) were packaged in 293T cells to express VSV-G protein as described in Materials and Methods. The TCID_50 _of VLPs was determined by titration in TZM-bl cells, and TERT-2 monolayers were then incubated for 6 h with VLPs at a MOI 10 (TCID_50 _per cell). Cells were then washed, trypsinized to remove unincorporated VLPs, and incubated for up to 48 h. Post inoculation, cells were fixed in 2% paraformaldehyde and nuclei were stained with DAPI (blue). (A) TERT-2 cultures were pre-incubated with AZT (0 to 2500 μM) before inoculation with VLPs. The expression of EGFP reporter gene was analyzed at 48 h post inoculation. (B) Kinetics of EGFP expression from 18 h to 48 h post inoculation. TERT-2 cells incubated with envelope-deficient particles were included as a negative control (48 h post inoculation). Arrows indicate EGFP expressing TERT-2 cells (green). Scale bar represent 50 μm. Images are representative of three independent experiments.

To demonstrate reverse transcriptase activity, we infected TERT-2 cells with pseudotype HIV-NL4-3Δenv-EGFP particles, which express the vesiculostomatitis virus glycoprotein (VSV-G) envelope (virus-like particles; VLPs). When infected, cells express EGFP as a reporter for HIV-1 LTR promoter activity and expression of viral-specific proteins. When TERT-2 cells were inoculated with VLPs at a MOI of 10, EGFP was expressed at a high level, confirming reverse transcriptase activity, LTR promoter activity and expression of new viral-specific proteins (reported by EGFP) (Fig. 2A). When the cells were pre-treated with increasing amounts of the viral inhibitor AZT (5 to 2500 μM), EGFP expression was inhibited in a dose-dependent manner (Fig. 2A). Integration and expression of HIV-1 specific proteins was stable since EGFP was expressed after 10 passages of TERT-2 cells (data not shown).

To estimate the kinetics of the HIV LTR promoter activity, EGFP expression was analyzed at indicated times post inoculation with VLPs (Fig. 2B). EGFP expression was first detected at approximately 18 h post inoculation and maximized at 48 h, reflecting the time course of activation of the HIV LTR promoter in infected cells.

To confirm HIV-1 integration in TERT-2 cells, we infected TERT-2 monolayers with HIV-1 strains IIIb or BaL and then performed a nested PCR with HIV- and human alu-specific primers (Table 2). These PCR reactions amplify HIV-1 sequences integrated in human genomic DNA. In preliminary experiments, we showed that laboratory stocks of HIV-1 are contaminated with DNA that is acquired from PBMCs during viral propagation (data not shown). The contaminating DNA was substantially resistant to DNase treatment of the HIV-1 stocks and could be amplified as a false-positive indication of integration. To eliminate contaminating sources on the plasma membrane or in the cytoplasm, integrated HIV-1 DNA was extracted directly from TERT-2 cell nuclei.

**Table 2:** Primer sequences and PCR conditions

**Target**	**Primer**	**Sequences (5'-3')**	**PCR conditions**
**Integrated HIV-1**			
**DNA^a^**			
**• First round PCR**	L-M667	ATGCCACGTAAGCGAAACTCTGGCTAACTAGGGAACCCACTG	95°C, 8 min and 95°C, 10 s, 60°C, 10 s, 72°C, 170 s for 12 cycles
	Alu 1	TCCCAGCTACTGGGGAGGCTGAGG	
	Alu 2	GCCTCCCAAAGTGCTGGGATTACAG	
**• Second round PCR**	Lambda T	ATGCCACGTAAGCGAAACT	95°C, 8 min and 95°C, 10 s, 60°C, 10 s, 72°C, 9 s for 40 cycles
	AA55M	GCTAGAGATTTTCCACACTGACTAA	
**Linear HIV DNA^a^**	MH531	TGTGTGCCCGTCTGTTGTGT	95°C, 8 min and 95°C, 10 s, 60°C, 10 s, 72°C, 6 s for 40 cycles
	MH532	GAGTCCTGCGTCGAGAGAGC	
**2-LTR circle^a^**	HIV F	GTGCCCGTCTGTTGTGTGTGACT	95°C, 8 min and 95°C, 10 s, 60°C, 10 s, 72°C, 10 s for 40 cycles
	HIV R	ACTGGTACTAGCTTGTAGCACCATCCA	
**U5-U3 RNA^a^**	HIV F	GTGCCCGTCTGTTGTGTGTGACT	95°C, 2 min and 95°C, 5 s, 60°C, 10 s, 72°C, 10 s for 40 cycles
	HIV R	ACTGGTACTAGCTTGTAGCACCATCCA	
**Gag**	For	CCCATAGTGCAGAACATCCA	50°C, 2 min, 95°C, 2 min, and 95°C, 15s and 60°C, 30s, for 50 cycles
	Rev	GGGCTGAAAGCCTTCTCTTC	
**Singly spliced^b^**	M669	GTGTGCCCGTCTGTTGTGTGACTCTGGTAAC	50°C, 2 min, 95°C, 2 min, and 95°C, 15s and 60°C, 30s, for 50 cycles
	La 23	GCCTATTCTGCTATGTCGACACC	
**Multiply spliced HIV RNA^a^**	P659	GACTCATCAAGTTTCTCTATCAAA	95°C, 4 min and 95°C, 5 s, 54°C, 10 s, 72°C, 8 s for 40 cycles
	P413MOD	AGTCTCTCAAGCGGTGGT	
**Unspliced HIV RNA^a^**	La 9	GACGCTCTCGCACCCATCTC	95°C, 2 min and 95°C, 10 s, 60°C, 40 s for 40 cycles
	La 8.1	CTGAAGCGCGCACGGCAA	
**β-actin**	Actin F	ATGGCCACGGCTGCTTCCAGC	95°C, 15 s, 55°C, 30 s, 72°C, 15 s for 30 cycles
	Actin R	CATGGTGGTGCCGCCAGACAG	
**GAPDH**	GAPDH F	GAGTCAACGGATTTGGTCGT	95°C, 15 s, 60°C, 30 s, 72°C, 15 s for 30 cycles
	GAPDH R	TTGATTTTGGAGGGATCTCG	

^a ^Primer sequences and PCR conditions were modified from [40]

^b ^Primer sequences and PCR conditions were modified from [74]

**Figure 3:**
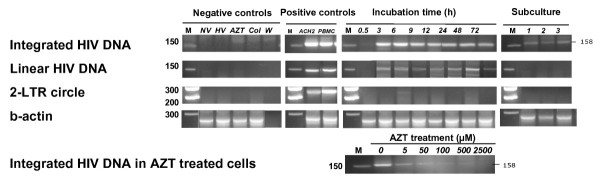
**Integrated HIV-1 DNA detected in TERT-2 nuclei**. TERT-2 cells were grown in monolayers and inoculated with HIV-1 (IIIb or BaL). Some cells were sub-cultured after infection. At 0.5 to 72 h post inoculation, TERT-2 cell nuclei were isolated as described in Materials and Methods. DNA was extracted from the nuclei and analyzed for integrated HIV DNA, linear HIV DNA, and 2-LTR circular HIV DNA. β-actin was included as a loading control. PCR reactions were performed as described in Materials and Methods (Table 2). Negative controls include cells without HIV-1 (NV), cells inoculated with heat-inactivated HIV-1 (HV), cells pretreated with 500 μM AZT (AZT), cells pretreated with colchicine (Col), and PCR reactions with no template (W). PBMC media (uninfected with HIV-1) and samples that were amplified in the second PCR only were also negative for HIV DNA (data not shown). ACH-2 cells and HIV-1-infected PBMCs served as positive controls for detection of integrated HIV DNA, linear HIV DNA, and circular HIV DNA. These agarose gel data for HIV-1 IIIb infection are representative of three independent experiments.

Infected TERT-2 nuclei contained integrated copies of HIV-1 from HIV strains IIIb and BaL, but only IIIb is shown (Fig. 3). Nested-PCR products were detectable in TERT-2 cell nuclei between 3 and 72 h post inoculation. An attenuated signal persisted after subculturing the cells for 1 to 3 passages, showing that integration is stable. Nuclei extracted from ACH-2 cells, an HIV_LAV _latent T cell clone [33], and HIV-infected PBMCs also contained HIV integrated DNA and served as positive controls. In contrast, integrated HIV-1 DNA was not detected in TERT-2 nuclei incubated in the absence of HIV-1 (NV), when HIV-1 was heat-inactivated (HV), or when cells were pre-treated with AZT (500 μM) or colchicine (500 μM).

From 3 to 72 h post inoculation, but not after subculture, total linear HIV DNA was detected in the nuclei of TERT-2 cells but not in the negative controls. Consistent with the low level of integration, HIV DNA two-LTR circles were not detected in TERT-2 cells except for a weak signal at 6 h post inoculation and not detected in the negative controls. When TERT-2 cells were pre-treated with increasing doses of AZT for 2 h followed by incubation with HIV-1 for 6 h, integration of HIV-1 DNA was inhibited. Results with BaL were similar (not shown).

### New HIV RNA transcripts in TERT-2 cells

Using RT-PCR, we attempted to detect new HIV-specific transcripts, including multiply spliced HIV-1 RNA, unspliced HIV-1 RNA, and U3-U5 HIV-1 RNA in TERT-2 cells. TERT-2 cells were incubated with HIV-1 IIIb or BaL for 6 h, trypsinized, washed, and incubated for up to 72 h. Some cells were sub-cultured after infection. Although we detected multiply spliced and unspliced products when using specific primers, these transcripts could not be distinguished clearly from contamination (data not shown). Multiply spliced and unspliced HIV-1 RNA appeared to degrade and were not detected after 12 h post inoculation. HIV-1 RNA species were also undetected after cells were sub-cultured and U5-U3 HIV-1 RNA was not detected at any time (data not shown).

New HIV-specific transcript levels were also estimated by SYBR real time RT-PCR relative to the level in the viral inoculum (data not shown). Relative to levels in the viral inoculum, multiply spliced and singly spliced HIV-1 RNAs were barely detectable.

**Figure 4:**
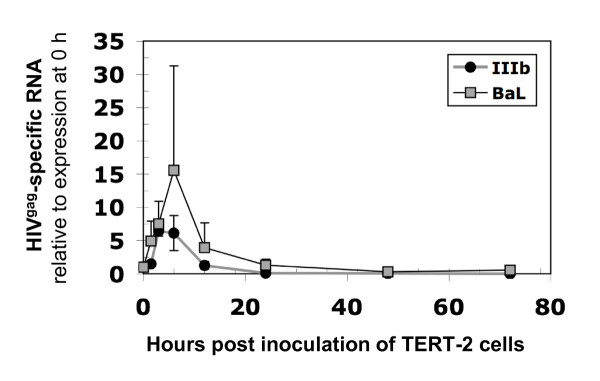
**HIV infection aborted in TERT-2 keratinocytes**. TERT-2 cell monolayers were incubated with HIV-1 (IIIb or BaL), trypsinized and washed. Cells were sub-cultured at 48 h post incubation. At 0.5 to 72 h post incubation, total RNA was isolated and cDNA was synthesized as described in the Materials and Methods. HIV^gag^-specific RNA was detected by SYBR real time PCR. β-actin served as the reference housekeeping gene. Data are the mean ± standard deviation of three independent experiments, each performed in triplicate.

HIV-1^*gag*^-specific RNA, however, was detectable. Using real time RT-PCR, HIV^*gag*^-specific RNA was quantified and the expression relative to 0 h was determined (Fig. 4). In TERT-2 cells, HIV^*gag*^-specific RNA appeared to increase up to 6 h post inoculation, suggesting that HIV-1 binds and enters TERT-2 cells. By 24 h post inoculation, however, the amount of HIV^*gag*^-specific RNA declined below the level of detection. If replication occurred, HIV-1^*gag*^-specific RNA was expected to increase during the 72 h incubation. The HIV-1^*gag*^-specific RNA decayed over time, however, and was not a product of new transcriptional events. In TERT-2 cells, therefore, RNA products of the HIV replication cycle were not prominent and replication appeared to abort.

### HIV-1 harboring in TERT-2 keratinocytes

**Figure 5:**
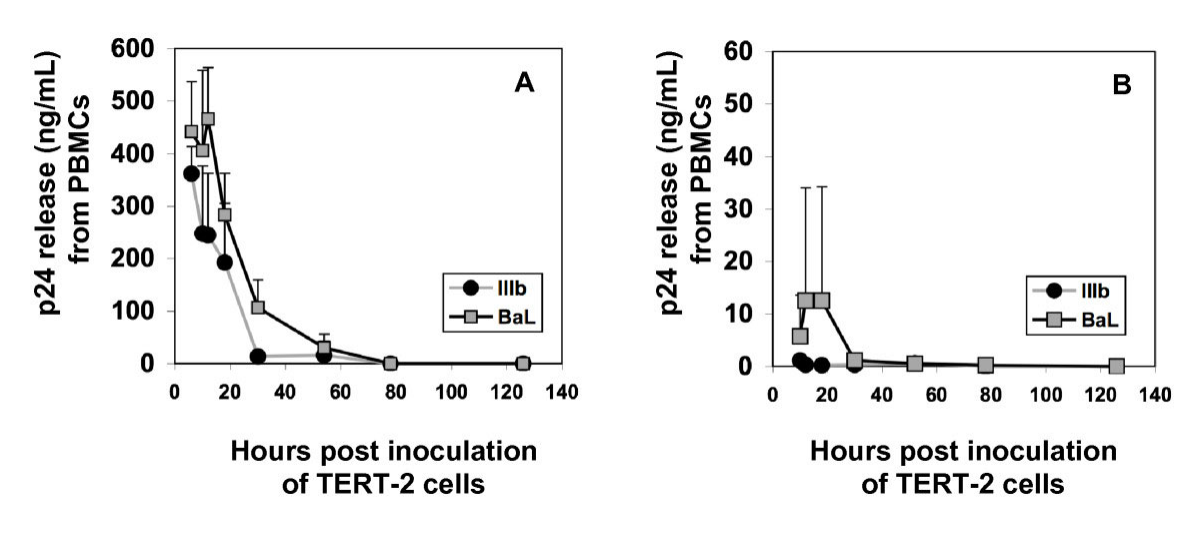
**Infectious HIV-1 harbored by TERT-2 cells**. TERT-2 monolayers were incubated for 6 h with HIV-1 (IIIb or BaL). TERT-2 cells were then trypsinized, washed, and maintained in growth media. At the indicated time post inoculation, TERT-2 cells were co-cultured with (A) PHA-activated PBMCs to test for direct transfer of HIV-1. To learn if infectious HIV-1 is released from TERT-2 cells, (B) spent media were recovered and used to inoculate PHA-activated PBMCs. After exposure to TERT-2 cells or media, PBMC supernatants were harvested at day 9 and analyzed for p24^gag ^production by ELISA. Data shown are the mean ± standard deviation from three independent experiments, each performed in triplicate.

To study harbored HIV-1, TERT-2 monolayers were incubated with HIV-1 for 6 h, then trypsinized, and washed to eliminate non-internalized viral particles. TERT-2 monolayers were maintained in culture for the indicated times up to 120 h post inoculation. To determine release of infectious virions, TERT-2 cell supernatants were aspirated (contains HIV-1 released from TERT-2 cells) and inoculated into PHA-activated PBMCs. After the infectious supernatants were aspirated, the TERT-2 cells were co-cultured separately with PHA-activated PBMCs to determine harbored virus available for direct transfer. TERT-2 cells appeared to harbor and *trans *infect X4- and R5 HIV-1 to permissive PBMCs (Fig. 5A). In contrast, supernatants from TERT-2 monolayers were barely infectious (Fig. 5B). HIV-1 *trans *infection by TERT-2 cells decreased to undetectable levels at 48 h (Fig. 5A and 5B), suggesting that harbored virus had decayed.

### MOLT-4/CCR5 cells acquire VLPs from infected TERT-2 cells

**Figure 6:**
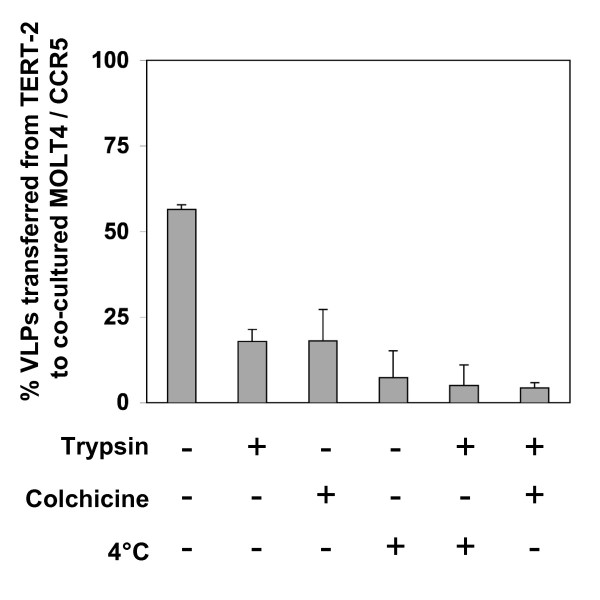
**TERT-2 cells *trans *infect VLPs to MOLT-4/CCR5 cells**. TERT-2 cell monolayers were incubated for 6 h at 37°C with replication-incompetent HIV-NL4-3 particles pseudotyped to express VSV-G envelope (VLPs) at a MOI 100. Cells were washed, then co-cultured with MOLT-4/CCR5 (2 × 10^5^) cells, and EGFP expression in MOLT-4/CCR5 cells was analyzed at 48 h using flow cytometry. The percentage of infected MOLT-4/CCR5 cells was quantified. Some TERT-2 monolayers were either treated with trypsin, colchicine (500 μM for 30 min), pre-cooled to 4°C, trypsin and pre-cooled to 4°C, or trypsin and colchicine as described in the Materials and Methods. Data shown are the mean ± standard deviation from three independent experiments.

To confirm that PBMCs acquire HIV-1 primarily by cell-to-cell interactions, TERT-2 cell monolayers were inoculated with replication-incompetent HIV-NL4-3Δenv-EGFP particles (VLPs) for 6 h at MOI 100. These VLPs replicate for a single round in the cell that ultimately becomes infected. TERT-2 cells with harbored, non-replicating HIV VLPs do not express EGFP. TERT-2 cells were then co-cultured with permissive MOLT-4/CCR5 T cells (MOLT-4/CCR5). VLP *trans *infection was determined by EGFP expression in MOLT-4/CCR5 at 48 h after co-culture. At 6 h post inoculation, TERT-2 cells captured and transferred 56% of the VLP inoculum to infect MOLT-4/CCR5 (Fig. 6). When TERT-2 cells were treated with trypsin at 6 h and washed to inactivate and remove extracellular virus, 18% of the VLP inoculum was transferred from within the TERT-2 cells to infect co-cultured MOLT-4/CCR5 cells. When TERT-2 cells were treated with colchicine (500 μM for 30 min before inoculation) to uncouple the tubulin cytoskeleton, 18% of the VLP inoculum was harbored and transferable to MOLT4 cells. At 4°C, 7% was harbored by untreated TERT-2 cells. A small percentage of VLPs were resistant to trypsin and colchicine treatments, infecting MOLT-4/CCR5 cells (4%) when co-cultured with TERT-2 cells. VLPs could bind TERT-2 cells at 4° or 37°C, but VLPs could be internalized efficiently only at 37°C suggesting that microtubule activity was required for internalization. The fraction of VLPs that resisted trypsinization and were sensitive to cold and colchicine appeared to be harbored within TERT-2 cells and transferred to MOLT4/CCR5 cells by direct cell-to-cell interactions.

## Discussion

Lining the oral and oropharyngeal mucosal surfaces, oral keratinocytes are potential targets for primary HIV-1 infection, harboring and dissemination. We now show for the first time that oral keratinocytes harbor and transfer viable HIV-1 to infect permissive cells for up to 48 h. Therefore, HIV-1 internalization by oral keratinocytes in vitro [18,20,22,34] may model an overlooked mechanism for HIV transmission and dissemination in vivo.

Although conventional wisdom suggests that primary human infection of permissive cells actually occurs in the gut [9,35], the oral mucosa in primate models becomes infected within a day of atraumatic oral mucosal exposure to SIV-1 [8,36]. Infection becomes marked in the GI tract four days after initial exposure, suggesting that virus disseminates from an oral focus.

Among oral mucosal sites, palatine tonsils are likely to disseminate HIV-1 since tonsil epithelial cells express appropriate receptors in situ [37,38], and *trans *infect HIV-1 to permissive cells in vitro. HIV *trans *infection in vitro from primary TE cells to PBMCs showed variation among tonsil donors. Since TE cells were derived from excised tonsils obtained with uncharacterized inflammatory backgrounds, proinflammatory cytokines might be differentially expressed in TE cells. Some cytokines may modulate HIV entry (reviewed in [39]), but whether donor-specific expression patterns affect primary infection is not known. Clearly, keratinocyte-associated virions remain infectious and can be transferred to infect co-cultured permissive cells.

Oral keratinocytes support the life cycle of HIV-1 step-by-step until integration. HIV^gag^-specific RNA peaked in TERT-2 cells after a 6 h incubation with HIV-1, which is consistent with the internalization of HIV-1 genomic RNA over time (Fig. 4; [40]). After internalization in TERT-2 cells, HIV-1 begins a replication cycle, which is not completed. HIV-1 genomic RNA is reverse transcribed into DNA, which can be inhibited by treatment of the cells with AZT (Fig. 2A). Among other reverse transcriptase products, linear HIV DNA was detected in oral keratinocytes, whereas two-LTR circles, stable forms of unintegrated HIV DNA, were not seen. This pattern of products is consistent with the low level of HIV-1 integration into TERT-2 cell genomic DNA.

To clarify the viral life cycle in TERT-2 cells, the presence of integrated HIV DNA was sought as a product of reverse transcriptase activity. Integrated HIV DNA was consistently detected in TERT-2 cell nuclei (Fig. 3). After incubation with HIV-1 in vitro, Liu et al. [20] had previously reported that oral keratinocytes contain linear HIV-specific DNA. We noted that contaminating DNA from the propagating cells is present in the viral inoculum and can be amplified by nested PCR, giving a false indication of integration. To avoid this artifact, we isolated HIV-1 DNA directly from the TERT-2 cell nuclei. As previously reported in permissive cells [40], integrated HIV DNA is detected consistently in TERT-2 nuclei and in all keratinocyte lines tested (data not shown) as early as 3 h post inoculation (Fig. 3). Integrated HIV DNA persisted in the TERT-2 genome after several passages of the cells, but the signal decayed for linear HIV DNA. To this point, replication kinetics in oral keratinocytes and permissive cells [40] were similar. The HIV-1 life cycle in TERT-2 cells was marked by viral internalization, uncoating, reverse transcriptase and integrase activities.

In response to infection by HIV-1 IIIb or BaL, the rate of decay of nonintegrated linear HIV-1 DNA in TERT-2 cells appeared to be too rapid to support substantial gene expression [40]. Likewise, we were unable to detect the specific RNA product U5-U3 RNA. HIV-1 specific mRNAs appeared at levels that could not be clearly distinguished from contamination (data not shown). After low-level integration, therefore, HIV-1 replication aborts.

Many steps in the HIV life cycle may be restricted by intrinsic cellular factors targeting viral entry, viral uncoating, viral DNA synthesis, intracellular trafficking of viral nucleic acids, integration, viral gene expression or viral packaging [41]. TERT-2 cells clearly restrict HIV replication after integration when infected with HIV-1. We sought to determine whether the internalization pathway used by HIV-1 in TERT-2 cells contributed to the restriction. Therefore we inoculated TERT-2 cells with VSV-G pseudotyped HIV-NL4-3Δenv-EGFP particles, which internalizes promiscuously into an endosomal pathway [42]. When integrated, HIV LTR from the pseudotyped particles regulates green fluorescence expression in TERT-2 cells (Fig. 3A and 3B). When the conventional, gp120-mediated viral entry is circumvented, the VSV-pseudotyped HIV-1 particles integrate and new RNA is transcribed. Since EGFP is expressed, viral-specific proteins are likely to be synthesized. This is in contrast to infection with the wild-type HIV-1 strains, where new transcripts are minimally expressed. Hence the CD4- and CCR5-independent internalization may represent a major restriction against HIV-1 replication.

The HIV entry mechanisms in oral keratinocytes and other epithelial cells are not well understood. Unlike oral keratinocytes, gastrointestinal epithelial cells constitutively express CCR5 and selectively internalize R5-tropic HIV-1 [43]. Oral keratinocytes from different sources are CD4- and express different putative receptors and co-receptors for HIV-1 including galactosylceramide [20] and heparin sulfate proteoglycans (HSPGs) [37,44-51]. HSPG binds HIV-1 gp120 [16,47,52-54], which can enter endosomes [55,56] and enable co-localization of HIV-1 particles with endosomal markers in TERT-2 cells (Dietrich E. et al, in preparation). Except for HSPGs, most putative receptors and co-receptors for HIV-1 are inconsistently expressed (Table 1) and can vary with the microanatomic location [37].

Although we saw no evidence of new HIV transcripts or newly replicated virions, TERT-2 cells clearly harbor infectious HIV-1 virions. Harbored HIV-1 can be effectively transferred to infect permissive cells including PBMCs for up to 48 h, but appear to become less infectious during the interval from 6 to 48 h after inoculation. After 48 h, TERT-2 cells were ineffective at *trans *infecting cell-associated harbored virus (Fig. 5A) and infectious supernatants (Fig 5B) to activated PBMCs. Since most experiments were performed after trypsinizing TERT-2 cells to remove extracellular virus, internalized HIV-1 was a harbored infectious reservoir.

HIV uptake and transfer are temperature and microtubule dependent (Fig. 6), as reported for endothelial cells [16]. With trypsin or colchicine treatment, harbored, internalized particles were distinguished from surface-bound particles. Both surface-bound and internalized particles are infectious and effectively *trans *infect CD4+ cells (Fig. 6). Cell-associated particles effectively *trans *infect PBMCs and MOLT-4 cells. Few infectious viral particles are released from TERT-2 cells. Optimal HIV transfer from TERT-2 cells is suggested therefore to involve direct cell-to-cell interactions with PBMCs and other permissive cells.

In the oral mucosa, the transfer of infectious virus to proximal lymphoid cells may be of clinical importance. Proximal to mucosal stratified squamous keratinocytes, Langerhans cells and CD4-positive lymphocytes are available to be *trans *infected in vivo. Indeed, a recent report suggests that Langerin-positive dendritic cells degrade internalized HIV-1, reducing transfer to CD4+ T cells in the mucosa [57], while others show that activated CD34-positive Langerhans cells increase *trans *infection of permissive target cells [58]. Unlike the female genital epithelium [17], oral Langerhans cells (dendritic cells) are not known to sample antigens or capture HIV-1 at the mucosal surface. Oral mucosal keratinocytes, therefore, could contribute to HIV transmission in vivo, however, by activating and *trans *infecting Langerhans cells, which can dock and transfer virus to CD4+ cells, or by transferring infectious harbored HIV-1 particles to proximal permissive cells.

For the first time, we show that oral keratinocytes become infected by HIV-1, initiating a defined, truncated viral life cycle. While infection is non-productive, an intracellular pool of infectious HIV-1 is harbored for up to 48 h and fully capable of *trans *infecting CD4+ permissive cells. Hence, the oral epithelium may actively disseminate HIV-1 infection and is more than an inert barrier. Since R5-tropic HIV-1 is most frequently associated with primary infections, oral epithelium could function as a selective "gatekeeper" and exclude X4-tropic virus. When compared, oral keratinocytes from different sources selectively harbor and transfer HIV-1 in either an X4- or R5-tropic HIV-1-specific manner (data not shown). TERT-2 cells consistently harbor all HIV-1 strains tested, while primary tonsil epithelial cells from some donors did not support *trans *infection (Fig. 1A). Since CXCR4+ CCR5- TERT-2 cells (Table 1) appear to harbor R5-tropic HIV-1 BaL more effectively than IIIb (Fig. 4), infection appears to be independent of the co-receptor tropism of the HIV envelope protein. We have recently shown that the endogenous oral pathogen, *Porphyromonas gingivalis*, selectively up-regulates CCR5 on CXCR4+ oral keratinocytes [59]. Up-regulation of CCR5 selectively promotes the harboring and transfer of R5-tropic HIV-1 from TERT-2 cells to permissive targets [60].

If oral mucosal keratinocytes serve as a clinical focus for HIV-1 infection, endogenous restriction factors notwithstanding, novel uptake, harboring and transfer mechanisms may become potential targets for antiviral drugs and vaccines. Following the initial short period of primary virus exposure, infectious HIV-1 persists in oral keratinocytes for several days. The harbored virus could be transferred to permissive cells and arguably serve to disseminate infection systemically. In the oral cavity, salivary components have been suggested to reduce the risk of HIV transmission [61-63]. For example, salivary mucins agglutinate the virus in vitro and appear to reduce viral uptake into permissive cells [64]. In the presence of saliva, however, HIV-1 still internalizes into oral keratinocytes in vitro and infectious virus can be effectively transferred to permissive reporter cells (Dietrich et al, 2008 in preparation). The rate of uptake of infectious HIV-1 into oral keratinocytes in the presence of saliva appears to occur more rapidly than complete inactivation of virus. Even in the presence of saliva, shedding oral epithelial cells may also serve as an infectious source for HIV transmission during oral sexual contacts. To protect against mucosal HIV transmission and dissemination, therefore, mucosal vaccines and microbicides should target the viral reservoir in oral keratinocytes.

## Conclusion

The oral mucosa is exposed to infectious HIV-1 during oral-sexual contact and breast-feeding. The surface oral and oropharyngeal epithelium is a potential site of primary HIV infection and dissemination even though these cells do not express the common HIV-1 receptors and co-receptors found on permissive cells. Using an atypical uptake mechanism (CD4-independent), oral epithelial keratinocytes were hypothesized to capture or internalize infectious HIV-1 and reverse transcribe the RNA HIV-1 genome into DNA, which then integrates into the keratinocyte genome. For the first time, integration, a major feature of infection, is shown to persist in daughter cells after the keratinocytes divide. After integration, the life cycle of the virus aborts and no newly assembled virus particles are detectable. By using HIV-1 that was engineered to bypass the usual receptors, we showed that the virus life cycle is prolonged. Although the life cycle aborts, captured infectious HIV-1 is harbored for at least 48 h and transferred to highly permissive peripheral blood mononuclear cells, which *in vivo *could result in systemic CD4+ T cell infection. While often considered passive bystanders in HIV-1 infection, mucosal epithelial cells could be actively providing a route to systemic infection.

## Materials and methods

### Cells

OKF6/TERT-2 immortalized keratinocytes (TERT-2), provided by Dr. James G. Rheinwald (Harvard Medical School, MA) were cultured in Keratinocyte-SFM (Invitrogen) supplemented (to final concentrations) with 0.2 ng/mL recombinant epidermal growth factor (rEGF; Invitrogen), 25 μg/mL bovine pituitary extract (BPE), and 0.4 mM CaCl_2_. Tonsil epithelial cells (TE) were isolated from tissue excised from HIV-seronegative individuals undergoing tonsillectomy at Hennepin County Medical Center, Minneapolis, MN. Use of surgical waste TE cells in research was reviewed and approved by the Research and Development Committee of the Minneapolis VA Medical Center and the Human Subjects Research Committee of the Hennepin County Medical Center. The protocol was determined to be exempt upon full IRB review and no subject consent was necessary. For culture, tonsillar epithelial cells were prepared by a modified method of Oda and Watson [65]. Briefly, tissue was cut and digested at 4°C overnight in 0.2% Dispase grade II (Boehringer Mannheim) in MEM supplemented with 10% FBS. The next day, epithelial sheets were separated from connective tissue, digested using 0.05% Trypsin/0.53 mM EDTA (GIBCO) at 37°C for 5 min, and dispersed into single cell suspensions using a pipette. Cells were cultured in keratinocyte-SFM supplemented with 5 ng/mL hEGF, 30 μg/mL BPE, and 0.06 mM CaCl_2_. For use in the experiments, TE cells from passage 3 or 4 were seeded at 10^4 ^cells/cm^2^. Molt-4/CCR5, ACH2, and TZM-bl cells were provided by the NIH AIDS Research and Reference Program. MOLT-4/CCR5 T cells were cultured in RPMI medium 1640 (Invitrogen) supplemented with 10% FBS and 1 mg/mL G418 sulfate. TZM-bl, and 293T were cultured in Dulbecco's Modified Eagle Medium (D-MEM; Invitrogen) containing 10% FBS. Peripheral blood mononuclear cells (PBMCs) were isolated from buffy coats obtained from 10 healthy seronegative donors by Ficoll-Histopaque density gradient centrifugation [66] and cryopreserved in liquid nitrogen until use. Source leukocytes from healthy adult donors were purchased from the Memorial Blood Centers. The Memorial Blood Centers IRB reviewed and approved the protocol. As part of the consent process, the blood donors agreed that their donated blood could be used for research purposes. When needed, PBMCs (2 × 10^6 ^cells/mL) were activated overnight in PBMC media (RPMI1640 medium containing L-glutamine (Mediatech, Inc.), 5% human interleukin-2 (Roche) with 10% FBS) supplemented with 5 μg/ml phytohemagglutinin (PHA-P; Sigma). After activation, cells were washed to remove PHA-P and cultured for 3 days in PBMC media before use.

### Viruses

HIV-1 strains IIIB (X4-tropic) and BaL (R5-tropic) were obtained from the NIH AIDS Research and Reference Reagent program. HIV-1 was propagated and TCID_50 _of virus stocks was determined in PHA-activated PBMCs as described in the Manual for HIV Laboratories, National Institutes of Health, Division of Acquired Immune Deficiency Syndrome (DAIDS) Virology (Publication NIH-97-3838).

### Virus-like particles

Plasmids encoding non-replicative NL4-3 (pNL4-3-Δenv-EGFP; Catalog number 11100) and the vesicular stomatitis virus G (VSV-G) glycoprotein (pHEF-VSV-G; Catalog number 4693) were obtained from NIH AIDS Research and Reference Reagent Program. To generate VLPs, 293T cells were transiently transfected with pNL4-3-Δenv-EGFP (10 μg) and pHEF-VSV-G (1 μg), using calcium phosphate precipitation as described previously [67]. To determine TCID_50 _of VLPs, TZM-bl cells (1 × 10^4 ^cells/well) were cultured overnight in 96-well tissue culture plates, and then incubated with six replicates of ten serial dilutions (1:4) of a VLPs stock in 50 μl growth media per well with the addition of 10 μg/mL Sequa-brene (Sigma). After 2 h, cells were washed three times and incubated in 200 μl of growth media. After 48 h, cells were fixed with 0.05% glutaraldehyde for 5 min at room temperature and washed twice with Dulbecco's phosphate-buffered saline (Mediatech, Inc.; DPBS). To detect the expression of β-galactosidase, cells were stained with 1 mg/mL X-Gal in 5 mM KFe_4_(CN_6_) 3H_2_O, 5 mM KFe_3_(CN_6_) 3H_2_O, and 1 mM MgCl_2 _and incubated at 37°C for 2 h. A positive well contained two or more blue cells. Positive- and negative-stained wells were tabulated and TCID_50 _was calculated using the Reed-Muench TCID_50 _calculation [68].

### Flow cytometry

TERT-2 or TE cells were washed once with DPBS and incubated with 0.02% (W/V) EDTA for 10 min. Detached cells were washed twice with DPBS supplemented with 2% FBS (wash buffer), and resuspended at 5 to 10 × 10^5 ^cells in 200 μL wash buffer. To identify putative HIV receptors and co-receptors, cells were incubated at 4°C for 30 min with 1 μg of anti-CD104, CXCR4, CCR5, galactosylceramide (GalCer), heparin sulfate (HSPGs), DC-SIGN, or macrophage mannose receptor (Table 1). Similarly, to characterize the purity of primary tonsil keratinocytes in culture, antibodies against CD3, CD4, CD11a/LFA1, CD32, CD64, CD89, and human fibroblast were used (Table 1). All antibodies were obtained from BD Pharmingen, except anti-GalCer (Chemicon), anti-heparin sulfate (Seikagaku) and anti-human fibroblasts (Sigma). Cells were then washed twice with 1 mL wash buffer to remove unbound antibody. If needed, cells were stained with goat anti-mouse IgG or IgM conjugated with fluorescein isothiocyanate (FITC) (Jackson ImmunoResearch Laboratories, West Grove, PA) in 200 μL wash buffer at 4°C for 30 min to detect primary antibodies. Isotype controls and other staining controls were included. After staining, cells were washed three times with 1 mL wash buffer, fixed in 200 μL of 2% paraformaldehyde, and stored at 4°C until analysis using a FACSVantage SE flow cytometer (BD Biosciences).

### HIV infection

To infect with HIV-1, TERT-2 cells were plated in 96-well tissue culture plates (1.5 × 10^4 ^cells/well) and grown overnight in monolayers to 80–90% confluence and infected at a MOI 0.01 (TCID_50 _per seeded cells), for 0.5 to 120 h. Every 48 h, media were replaced with fresh growth media to maintain viability of TERT-2 and TE cells. In some experiments, viruses were heat-inactivated (HV) by incubating in a water bath at 70°C for 3 h and used as a negative control. At indicated times, HIV-1 was aspirated. To remove surface-bound HIV-1, some cultures were treated with 0.05% trypsin/0.53 mM EDTA for 3 min at room temperature, and then an equal volume of soybean trypsin inhibitor (250 μg/mL; Invitrogen) in HBSS was added. Trypsinization did not appear to disrupt the monolayers, which were washed three times in HBSS and maintained in growth media. Some cells were sub-cultured for 3 to 8 passages post inoculation. In some experiments, cells were pre-treated with azidothymidine (AZT; 500 μM; Sigma) for 2 h, or colchicine (500 μM; Sigma) for 30 min and then inoculated with HIV-1. Colchicine was washed from cultures before HIV-1 was added, but AZT remained with TERT-2 cells during HIV incubation. To determine if reagent carry over inhibited replication in permissive cells, colchicine or AZT was incubated with TERT-2 cells. The treated and untreated TERT-2 cells were co-cultured with MOLT-4/CCR5 cells and VLPs (see below). Infectivity of VLPs (EGFP expression) in MOLT-4/CCR5 cells was similar when co-cultured with TERT-2 cells in the presence or absence of AZT, suggesting that contamination from TERT-2 cell cultures was insufficient to inhibit infection in permissive lymphoid cells.

### PBMC co-culture assays

At indicated times post inoculation, TERT-2 cells were co-cultured in triplicate wells of 96-well plates with 2 × 10^5 ^activated PBMCs to estimate *trans *infection of cell-associated HIV. Co-culture was performed in 200 μL of PBMC medium, which selectively supports viability of the PBMCs at the expense of the TERT-2 cells (require K-SFM supplement as above). After co-culture with HIV-infected TERT-2 cells, PBMC media were replaced (100 μL) on day 4, and supernatants were collected (100 μL) on day 9 by centrifugation at 330 × g for 5 min. The recovery of p24^gag ^was estimated in the PBMC supernatants with the Coulter HIV-1 p24^gag ^Antigen Assay (Beckman Coulter) using the manufacturer's protocol. To estimate the release of HIV-1 from TERT-2 cells, TERT-2 culture supernatants (50 μL) were collected and then inoculated into 2 × 10^5 ^activated PBMCs at selected times post inoculation. p24^gag ^production was estimated in PBMCs cultures nine days later as described above.

### Identification of integrated HIV DNA, linear HIV DNA and two-LTR circles

To identify integrated HIV DNA in TERT-2 cells, contaminating DNA from viral inocula (MOI 0.01) derived from propagating cells was carefully excluded. To partition contaminating DNA copies from TERT-2 cell integrated HIV DNA, TERT-2 cells (9 × 10^4 ^cells) were grown in 6-well plates and nuclei were isolated using the Nuclei EZ Prep Nuclei Isolation kit (Sigma). DNA was then extracted from the nuclei using the DNeasy kit (Qiagen) and quantified spectrophotometrically. PCR reactions contained 500 ng of TERT-2 DNA, primers and PCR conditions were as described (Table 2; [25,40]). Integrated HIV DNA was detected by nested PCR to increase sensitivity and fidelity [25]. PCR products were identified on 3% agarose gels stained with ethidium bromide.

### Analysis of multiply spliced, singly spliced, unspliced and U5-U3 HIV-1 RNA

Total RNA was collected from infected TERT-2 cells using Rneasy Plus Mini kit (Qiagen) and quantified spectrophotometrically. To detect viral-specific RNA using real time RT-PCR, 5 μg of total RNA was reverse transcribed to cDNA using an Iscript™ cDNA Synthesis Kit (BioRad). In separate PCRs, the cDNA product (10 μl) was incubated with primers specific to multiply spliced HIV RNA, unspliced HIV RNA and U5-U3 RNA. Primer sequences and PCR conditions were as shown (Table 2; [25]). Glyceraldehyde-3-phosphate dehydrogenase (GAPDH) sequence was amplified as a control. PCR products were identified on 3% agarose gels stained with ethidium bromide. The concentration and purity of RNA preparations was performed using the 2100 Bioanalyzer (Agilent). Total RNA (500 ng) was reverse transcribed to cDNA using the Superscript III First Strand Synthesis System. The cDNA was then diluted 1:5 with RNase/DNase free water and 1 μl (5 ng) was used as a template in the Platinum SYBR Green qPCR SuperMix-UDG with ROX (Invitrogen). Real time PCR was performed on each sample in triplicate on an ABI7900 HT Real Time PCR machine (Applied Biosystems) and data was analyzed using SDS 2.1 software (Applied Biosystems). All genes were normalized to expression of human β-actin (SuperArray Bioscience). Relative expression was quantified using the delta-delta CT method [69].

### VLP infection

To prepare for infection with virus-like particles (VLPs; pseudovirus), cells were grown overnight on gelatin-coated cover slips in 24-well plates to approximately 50% confluence. Cell monolayers were then incubated with VLPs at a MOI 10 for 6 h with 10 μg/mL Sequa-brene and then aspirated. At indicated times post inoculation, cells were fixed in 4% paraformaldehyde at room temperature for 10 min, washed three times in 1 mL DPBS, and nuclei were stained with 4', 6-diamidino-2-phenylindole, dihydrochloride (DAPI; Molecular Probes). Cells were then washed three times in DPBS and the glass cover slips were mounted with Fluoromount G (Southern Biotech). EGFP expression was visualized with a fluorescence microscope (Eclipse E800, Nikon) under a 20× objective. Images were acquired using Spot Insight QE (Diagnostic Instrument, Inc.) and MetaMorph software (Molecular Devices). To characterize *trans *infection, TERT-2 cultures in 24-well tissue culture plates were incubated with VLPs at a MOI of 100 for 6 h in the presence of 10 μg/mL Sequa-brene. Supernatants were aspirated and TERT-2 cells were co-cultured with MOLT-4/CCR5 (2 × 10^5^) cells. In some experiments, TERT-2 cells were treated with trypsin or colchicine as above, or cooled to 4°C (or combinations of treatments) and transfer of VLPs was compared. At 48 h after co-culture, MOLT-4/CCR5 cells were collected by centrifugation at 330 × g for 5 min, and washed three times in 1 mL DPBS with 2% FBS. Cells were then resuspended in 200 μL of 2% paraformaldehyde, and stored at 4°C. EGFP-positive cells were analyzed by use of a FACSVantage SE flow cytometer (BD Biosciences).

## Competing interests

The authors declare that they have no competing interests.

## Authors' contributions

AV contributed to the design of the study, evaluated the data, drafted the manuscript and performed all of the experimental procedures except as noted. AA carried out the SYBR real time PCR assays. KG prepared HIV stocks. CF performed flow cytometric analyses. RG and all authors contributed to the critical appraisal of the data. EJ contributed to the early design of the study. KR and MH conceived of the study, contributed to the design and coordination of the experiments, and critically reviewed and edited the manuscript. All authors read and approved the final manuscript.
